# Supplementary figures and images for: Microbial corrosion of DSS 2205 in an acidic chloride environment under continuous flow
Source: PLoS One. 2021 May 12;16(5):e0251524. doi: 10.1371/journal.pone.0251524 (PMC8115847; doi:10.1371/journal.pone.0251524)

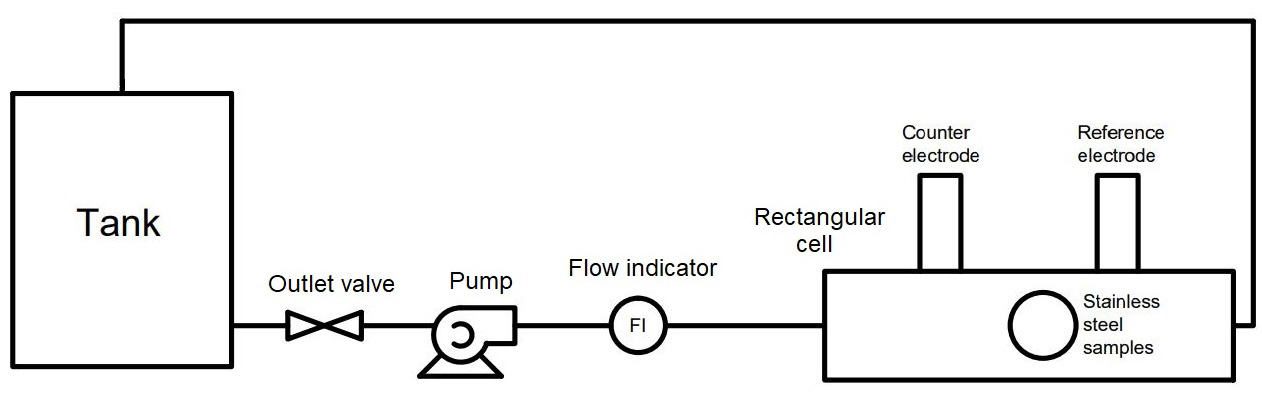

Supplement: S1 Fig — (TIF) [file pone.0251524.s001.tif]

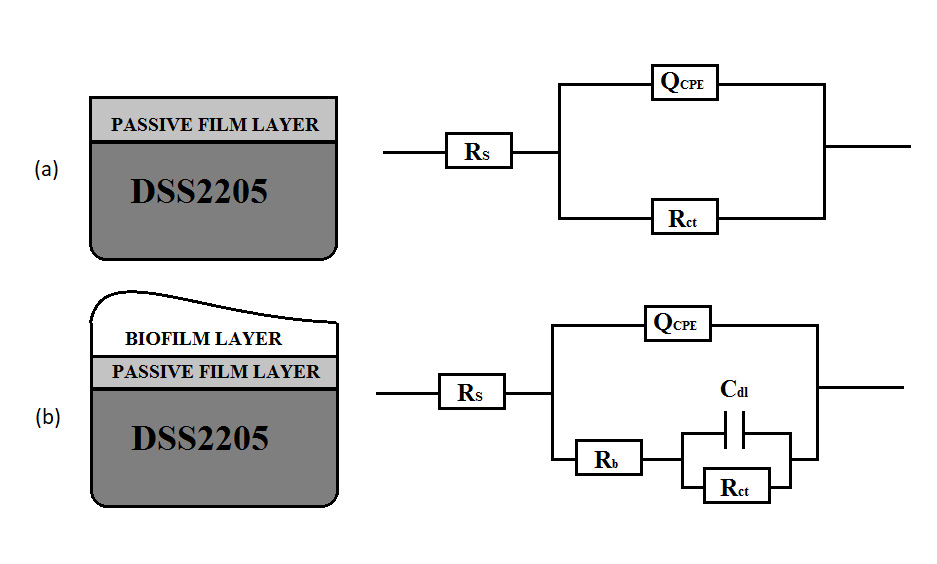

Supplement: S2 Fig — (TIF) [file pone.0251524.s002.tif]
