# Supplementary material for: Microbial corrosion of DSS 2205 in an acidic chloride environment under continuous flow
Source: PLoS One. 2021 May 12;16(5):e0251524. doi: 10.1371/journal.pone.0251524 (PMC8115847; doi:10.1371/journal.pone.0251524)
Supplement: Attachment — Submitted filename: Response to Reviewers.docx [file pone.0251524.s003.docx]

**Response to reviewers’ comments**

*Dear reviewers,*

*Thank you for giving us the opportunity to submit a revised draft of our manuscript. We appreciate the time and effort that you have dedicated to providing the valuable feedback on our manuscript. We are grateful to you for your insightful comments on our paper. We have been able to incorporate changes to reflect the suggestions provided by the reviewers. We have highlighted the changes within the manuscript.*

**Reviewer 1:**

The manuscript entitled “Microbial corrosion of DSS 2205 in an acidic chloride environment under continuous flow” attempted to assess the influence of bacteria Desulfovibrio vulgaris on the corrosion of stainless steel coupons DSS 2205 at different speeds of seawater flow under microcosm conditions. The manuscript is interesting since the subject has a direct impact on the corrosion of DSS 2205 in a condition that has only been addressed for a few years, despite being a major factor in the formation of corrosive biofilms.
I believe that the manuscript is worthy of being published in the journal PlosOne. I have few considerations:
In the introduction, the citation number 5, although important, is too old, currently, new considerations are addressed in the reviews, so I suggest that they be cited:
Procópio L (2020) The era of 'omics' technologies in the study of microbiologically influenced corrosion. Biotechnol Lett 42 (3): 341-356.

**Response: The recommended reference has been added to the manuscript as reference number 4.**

Approaches to the role of corrosion in oil facilities should be cited:
Procópio L (2020) Changes in microbial community in the presence of oil and chemical dispersant and their effects on the corrosion of API 5L steel coupons in a marine-simulated microcosm. Appl Microbiol Biotechnol 104 (14): 6397-6411.
**Response: This reference has been added as reference number 5.**

Still in the introduction, in lines 42-44 I suggest citing the publications below. They constitute the few works that have addressed the topic since the manuscript, and should be considered:
Trueba A, Eguía E, Milad MM (2010) Biofouling growth on tubular heat exchangers. Mathematical model and simulation. J Maritime Res 8: 15–34.
Trueba A, García S, Otero FM, Vega LM, Madariaga E (2015) Influence of flow velocity on biofilm growth in a tubular heat exchanger-condenser cooled by seawater. Biofouling 31: 527–534.
Tsai YP (2005) Impact of flow velocity on the dynamic behavior of biofilm bacteria. Biofouling 21: 267-277.
**Response: These references have been added as references 17-19.**

In terms of material and methods, were the analyzes about the coupons used, working as electrode or biofilm analysis, not carried out in triplicate? It is not clear the replication of the experiment as a whole.
**Response: The experiments were performed in triplicate. In each experiment, there were two coupons used in the experiment, one coupon was used as working electrode and one coupon was used for biofilm observation. The purpose of using a separate coupon for biofilm observation is to avoid the biofilm damage caused by the applied voltage on the working electrode.**

I believe that a quantitative estimate of the number of cells present on the coupons would be relevant to validate the differences in biofilms in both conditions. CFU or qPCR techniques can answer this question.

**Response: During the experiments, only the number of bacterial cells in solution were counted using haemocytometer to observe the growth of bacteria and to ensure to have a suitable number of bacteria in the solution (around > 10^4^ cells/mL) (data not shown). The differences in the biofilms were studied qualitatively by observing the coverage of the coupons surfaces by optical microscopy (OM) and scanning electron microscopy (SEM). Unfortunately, we did not perform CFU and qPCR techniques.**

**Reviewer 2:**

The authors investigated the acidic flow for biotic and abiotic corrosion of stainless steel. While the importance of the knowledge of acidic flow is not very clear, the observation shown in the flow system is reasonable, and should be published. I have a few questions regarding the observation.
1. D. vulgaris is anaerobic bacteria and how they could grow under aerobic flow condition?

**Response: The experimental work in the manuscript was conducted in a closed-circuit anaerobic condition. The tank, the pipe and the solution were flushed with nitrogen gas before the experiment. The top of the tank was also covered with a layer of nitrogen gas to create an anaerobic condition inside.**

3. oxygen scavenger conc dependency would be interesting experiments to add.

**Response: Thank you for your suggestion. We will aim to include such condition in further research to improve the literature.**

2. Biofilm formation can be labeled by fluorecent dye, and such experiment should be done.

**Response: The experiment was performed in triplicate. In the first experiment, the biofilm formed on the coupon stained with DAPI and observed under fluorescence microscope (photos not shown as low quality) and observed under optical microscope. We found that under OM (optical microscopy), we can see the biofilm formation more clearly and these images were used for the manuscript.**

4. Too many Figure numbers. Some of them can be unified.

**Response: The number of figures was reduced to 8 figures. Fig. 1 and Fig. 8 were moved to a supplementary file, Fig 2 and 3 were changed to Fig 1(A) and 1(B).**
